# Keystone Veterinary Medical Association

**Published:** 1894-12

**Authors:** W. L. Rhoads

**Affiliations:** Secretary


### KEYSTONE VETERINARY MEDICAL ASSOCIATION.

The December meeting of the Keystone Veterinary Medical Association was held at 3452 Ludlow Street, Philadelphia, on the evening of December 11th. President Lintz called the meeting to order. On roll call by Secretary Rhoads, the following members responded to their names: Drs. Bridge, Cullen, Hoskins, Lintz, Jno. R. Hart and Rhoads. As visitors, Drs. Thos. B. Rayner, F. S. Allen, H. T. George and J. T. McAnulty.

The report of the Board of Trustees was called for, and they reported the financial standing of the members now carried on the roll. After further discussion on the subject, the Secretary was instructed to proceed to collect the various sums of indebtedness of the members, and report at the January meeting.

The special Committee to appoint essayists for the various meetings, and those who were to report cases, was adopted, and the Secretary was instructed to notify these members of their appointments.

A revised bill looking to the establishment of a Live Stock Sanitary Commission in Pennsylvania was then presented, together with an appeal for support of the same, and after some discussion the Association indorsed the bill.

The subject of "Azoturea," which had been assigned for this meeting, was then considered. and cases were reported by Drs. Bridge. Allen, Cullen, T. B Rayner, McAnulty, Lintz and Hoskins. The value of purgatives was discussed, and a difference of opinion as to their value was expressed. The need of some remedies to produce diaphoresis was urged on the grounds that we always find the skin attempting to assist the kidneys in the eliminating from the body effete products which are essential to a successful termination of the case, and, as it always seems to act more promptly than the bowels, its assistance seems to be indicated as a very desirable means of a more successful ending of these cases.

Dr. Jas. T. McAnulty's application for associate membership was received and was referred to the Board of Trustees to report at the January meeting.

Some general discussion followed, after which the meeting adjourned.

W. L. Rhoads, Secretary.

---

